# Selection and Validation of Reference Genes for Gene Expression Analysis in *Tuta absoluta* Meyrick (Lepidoptera: Gelechiidae)

**DOI:** 10.3390/insects12070589

**Published:** 2021-06-29

**Authors:** Xin Yan, Yibo Zhang, Kangkang Xu, Yawei Wang, Wenjia Yang

**Affiliations:** 1 Guizhou Provincial Key Laboratory for Rare Animal and Economic Insect of the Mountainous Region, College of Biology and Environmental Engineering, Guiyang University, Guiyang 550005, China; yanxin@126.com (X.Y.); kkxu1988@163.com (K.X.); gywangyawei@163.com (Y.W.); 2 State Key Laboratory for Biology of Plant Diseases and Insect Pests, Institute of Plant Protection, Chinese Academy of Agricultural Sciences, Beijing 100193, China

**Keywords:** tomato leaf miner, reference gene, development, 20-hydroxyecdysone, insecticide, gene expression, RT-qPCR

## Abstract

**Simple Summary:**

Reference genes are critical for standardizing expression data of RT-qPCR across samples of organisms under different experimental conditions. However, most commonly used reference genes may not be stably expressed leading to a risk of misinterpretation of results. In our study, nine reference genes were evaluated in *Tuta absoluta* (a destructive pest of tomato) at different developmental stages, tissues, 20-hydroxyecdysone (20E) and insecticide treatments. Finally, the expression profile of indicator gene *EcR* after 20E treatment was evaluated to verify the accuracy of the results. This study is essential for improving accuracy and reliability to normalize gene expression data in *T. absoluta* and provides a useful strategy for other insects.

**Abstract:**

The tomato leaf miner, *Tuta absoluta* is a destructive pest of tomato. The leaf-mining activities of its larvae can cause significant yield losses. Real-time quantitative polymerase chain reaction (RT-qPCR) is commonly used to measure gene expression, and the selection of stable reference genes for calibration and standardization is critical for accurate use of RT-qPCR. We studied the stable expression of nine common housekeeping genes in *T. absoluta*. These were examined at different developmental stages, in larval tissues, as well as those induced by exposure to 20E and insecticides. Four dedicated algorithms (geNorm, BestKeeper, NormFinder, and ΔCt method) and online tool (RefFinder) were used to analyze and rank the tested reference genes. Based on the standardized gene expression data of target gene *ecdysone receptor* (*EcR*), the applicability of specific reference genes was verified. The results clarify that the optimal internal reference genes vary greatly under different experimental conditions. *GAPDH* and *RPS11* were the best reference genes for developmental stages; *RPL28* and *RPL10* for different tissues; *EF1α* and *RPL28* for 20E treatment; *EF1α* and *RPL7A* for insecticide treatments. The most suitable reference genes in all experimental conditions are *EF1α* and *RPL28*.

## 1. Introduction

Real-time quantitative polymerase chain reaction (RT-qPCR) is a powerful tool for nucleic acid quantification due to its sensitivity, specificity, and reproducibility [1]. However, the accuracy and reliability of RT-qPCR results are affected by several factors, including the quality and quantity of the initial RNA, primer specificity, reverse transcription efficiency, amplification efficiency, PCR conditions, reference genes, and transcript standardization [2,3]. For accurate quantification, stable reference genes are necessary for normalization [4]. The reference genes used in an RT-qPCR experiment can significantly influence the calculation of the expression levels of target genes [5].

Due to the temporal and spatial specificity of gene expression, the stability and effectiveness of internal reference genes are limited to specific conditions [3,6]. Therefore, the reference genes usually chosen include housekeeping genes that have stable expression in different cells or during different physiological states [7]. *Elongation factor* *1-*α (*EF1α*), *glyceraldehyde-3-phosphate dehydrogenase* (*GAPDH*), and *β*-actin (*ACT*) are constitutively expressed genes that are commonly used as reference genes [8,9]. However, it is unclear if the expression of these genes are always stable. There is no single “universal” reference gene [10]. To obtain accurate results, the exact experimental conditions for the expression of each candidate reference gene must be verified to avoid the ambiguity of data in RT-qPCR analysis [11].

The stability of reference genes has been studied in many lepidopteran insects. For example, *GAPDH* was found to be the most appropriate reference gene for different developmental stages and tissues in *Thitarodes armilicanus* [12]. In *Diaphania caesalis*, *ACT* and *60S ribosomal protein L13a* (*RPL13a*) were the most stable reference genes in different developmental stages, *ACT* and *eukaryotic initiation factor 4A* (*EIF4A*) were recommended as stable reference genes in various tissues [13]. Three genes, *18S ribosomal RNA* (*18S*), *ribosomal protein S20* (*RPS20*), and *α-tubulin (α-TUB)*, were most suitable for gene expression in fifth-instar larvae of *Sesamia inferens* exposed to temperature stresses [14]. The stability of reference genes can greatly differ in the same insect under different conditions. Two or more reference genes may be necessary for some species, as a single reference gene cannot satisfy experimental requirements [9,11].

The tomato leaf miner, *Tuta absoluta* Meyrick (Lepidoptera: Gelechiidae), is a worldwide pest native to Peru. It occurs in South America, Europe, Africa, and Asia [15,16]. Recently, it was discovered on open-field tomatoes in Ili, Xinjiang Uygur Autonomous Region of China [17]. *T. absoluta* mainly feeds on the leaves of *Solanaceous* plants such as tomatoes, potatoes, and eggplants. It also feeds on fruits, apical buds, tender shoots, and tender stems, and can greatly reduce crop yield [15]. Although the requirements for accurate verification of reference genes in RT-qPCR studies have been described, the normalization procedure for *T. absoluta* has not been reported. Our goal was to identify endogenous reference genes that are suitable for gene expression profile analysis under a variety of experimental conditions. Nine commonly used reference genes, *arginine kinase* (*AK*), *superoxide dismutase* (*SOD*), *ribosomal protein L7A* (*RPL7A*), *ribosomal protein* *L10* (*RPL10*), *ribosomal protein* *L28* (*RPL28*), *ribosomal protein S11* (*RPS11*), *EF1α,* *GAPDH, and* *ACT* were selected for analyzing their expression stability under different experimental conditions in *T. absoluta*. Four dedicated algorithms (geNorm, NormFinder, BestKeeper, and ΔCt method) were used to analyze quantifiable data [18]. The online tool RefFinder combines the above-mentioned algorithms to compare and ranked the tested reference genes. RefFinder also assigns a suitable weight to each gene and calculates the geomean of the weights to provide an overall ranking [19]. Finally, the expression profile of *EcR* was evaluated after 20E treatment to validate the results.

## 2. Materials and Methods

### 2.1. Insect Rearing

*Tuta absoluta* was originally collected from *Solanum lycopersicum* in Yuxi city, Yunnan Province, China, and reared for more than ten generations in the laboratory. The larvae were reared on tomato plants, where fed on leaves. The adults were fed with 10% honey solution in a greenhouse. The colonies were kept at 26 ± 1 °C under 60 ± 5% humidity and a 16:8 h light/dark (L:D) photoperiod.

### 2.2. Biotic Factors

Different developmental stages of *T. absoluta* were prepared. The insect culture was initiated with uniform aged eggs to collect insects in different, but uniform developmental stages, including the first-, second-, third-, fourth-instar larvae, pupae, and adults (including both males and females) of *T. absoluta*. All samples were immediately frozen in liquid nitrogen for RNA extraction. Three biological replicates were collected for each stage.

Seven tissues, including head, integument, fat body, foregut, midgut, hindgut, and Malpighian tubule, were dissected from the third-instar larvae in pre-cooled PBS solution [8,11]. The total RNA for each sample was extracted as described below.

### 2.3. Abiotic Stresses

The leaf-dip bioassay method suitable for *T. absoluta* was slightly modified from a previous report [20,21]. We dissolved 4 mg 20E (Sigma-Aldrich, St. Louis, MO, USA) in 95% ethanol as the stock solution with a concentration of 10 μg/μL, and then were diluted to 1 μg/μL with distilled water. We selected third-instar larvae with uniform size and good health for continued feeding following exposure to 20E solution by leaf immersion while feeding 0.1% ethanol as a control. Treated larvae were reared under the same conditions as the stock insects. At 12, 24, 48, and 72 h after treatment, the whole bodies of surviving insects were randomly selected and frozen in the liquid nitrogen for RNA extraction.

The insecticides used were abamectin, spinosad, chlorantraniliprole, and indoxacarb, as these are commonly used for the control of lepidopteran pest [22,23]. Tomato discs of 3 cm diameter were cut and then dipped in different concentrations of insecticides containing 0.1% Triton X-100. Each disc was immersed for 10 s and allowed to air dry at room temperature for three minutes. The discs were then individually placed into plastic Petri dishes. A total of 20 third-instar larvae were transferred into each dish, and three biological replicates were conducted. Tomato discs treated with the 0.1% Triton X-100 without insecticides were used as controls. All the larvae were reared under normal conditions, and mortality was checked after 24 h. The 50% lethal dose (LD_50_) values for each insecticide were assessed by probit analysis after 24 h. The third-instar larvae were then treated with the LD_50_ dosage of each insecticide. The surviving larvae were collected 24 h after the insecticide treatment and then frozen in the liquid nitrogen before RNA extraction.

### 2.4. Total RNA Extraction and Reverse Transcription

Total RNA was extracted from each sample using MiniBEST Universal Extraction Kit (TaKaRa, Dalian, China). RNA quantity were measured by a NanoDrop 2000c spectrophotometer (Thermo Fisher Scientific, Waltham, MA, USA) with absorbance levels of 260 nm and 280 nm. RNA integrity was checked by 1.0% agarose gel electrophoresis. One microgram of RNA was used to synthesize the first-strand cDNA by TransScript Synthesis Supermix (TransGen Biotech, Beijing, China). The concentration of all cDNA samples was normalized to 500 ng/μL. The cDNAs were stored at −20 °C for later RT-qPCR experiments.

### 2.5. Candidate Reference Genes and Primer Design

Based on the *T. absoluta* transcriptomic data (SRR13065833), nine commonly used reference genes were selected, including *EF1α*, *AK*, *SOD*, *RPL7A*, *ACT*, *GAPDH*, *RPL10*, *RPL28*, and *RPS11*. The functions of these genes are listed in Table 1. The primers were designed using NCBI Primer-BLAST (https://www.ncbi.nlm.nih.gov/tools/primer-blast/, accessed on 15 March 2021), and primer sequences are listed in Table 2.

### 2.6. RT-qPCR Analysis

The RT-qPCR was carried out on a CFX-96 real-time PCR system (BioRad, Hercules, CA, USA) using 20 μL reaction mixtures containing GoTaq^®^ qPCR Master Mix (Promega, Madison, WI, USA), 10 μM each gene-specific primer, and cDNA template. The reaction conditions were as follows: an initial denaturation at 95 °C for 5 min, followed by 40 cycles at 95 °C for 5 s, 55 °C for 30 s, and 72 °C for 30 s. A melting curve step cycle (55 °C for 10 s, and then 0.5 °C for 10 s until 95 °C) followed the amplification and was added to ensure the specificity of the primers. Three technical replicates were run for each biological replicate. One unique peak in the melting curve confirmed the gene-specific amplification in each pair of primers. The quantitative data for each gene was analyzed for the slopes with a linear regression model. Standard curves were generated based on a 4-fold dilution series of cDNA (1/4, 1/16, 1/64, 1/256, and 1/1024). The corresponding amplification efficiencies were calculated according to the equation: E = (10[−1/slope] − 1) × 100%.

### 2.7. Stability of Gene Expression

The expression stability of each candidate reference gene was evaluated by geNorm, NormFinder, BestKeeper, and ΔCt method, followed by a comprehensive ranking by RefFinder (http://www.ciidirsinaloa.com.mx/RefFinder-master/ accessed on 15 March 2021). The optimal number of reference genes for target gene expression normalization was determined by pair-wise variation (V_n_/V_n+1_) using V-values that were calculated using geNorm. A V_n_/V_n+1_ cut-off value of ≤0.15 signified that the additional n+1 reference gene was unnecessary and confirmed the appropriate number of reference genes for RT-qPCR data normalization [24].

### 2.8. Validation of Selected Reference Genes

The ecdysone receptor (*EcR*) of *T. absoluta* was selected as the target gene for stability validation. The relative expression of *EcR* in the 20E treatment was quantified according to threshold cycle (Ct) value by the 2^−ΔΔCt^ method [13]. Differences in gene expression were compared using SPSS 20.0 software (IBM, Armonk, NY, USA) (ANOVA, LSD method). All data were visualized using the GraphPad Prism version 8.0.1 (GraphPad Software, La Jolla, CA, USA) and presented as mean ± standard error.

## 3. Results

### 3.1. Total RNA Quality and Amplification Efficiencies

The A260/280 ratios ranged from 1.81 to 2.25, indicating the high purity of all the RNA samples. Gene-specific amplification of nine genes was verified in *T. absoluta* larvae by a single, sharply defined peak in the melting curve analysis, suggesting a high level of specificity (Figure S1). The RT-qPCR efficiency ranged from 92.9% (*RPL28*) to 109.1% (*SOD*), and regression coefficient ranged from 0.991 (*ACT*) to 0.998 (*AK* and *RPL10*) (Table 2). The results indicated that all primers met the standard requirement for RT-qPCR analyses [6].

### 3.2. Expression Profiles of Nine Candidate Reference Genes

**Figure 1:**
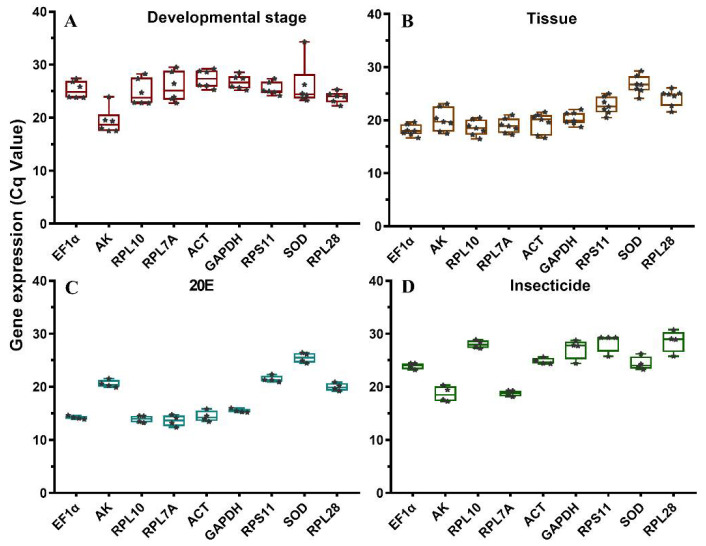
Box and whisker plots of expression profiles of candidate reference genes under four experimental conditions. (**A**) Different developmental stages; (**B**) different tissues; (**C**) 20E treatment; (**D**) insecticide treatments. The expression levels of candidate reference genes are shown as the mean of Ct values. The Ct values of each biological replicate in each treatment is represented as each data points. Lines across the boxes depicted the medians of Cq values, and the 25/75 percentiles were indicated by the lower and upper lines of boxes.

The expression level was determined as the number of cycles required for amplification to reach the threshold (500) in the exponential phase of the PCR reaction [17]. The overall threshold cycle (Ct) values were compared to evaluate the expression levels of reference genes under different experimental conditions. The raw Cq values ranged from 17.49 (*AK*) to 34.3 (*SOD*) for the various developmental stages, from 16.67 (*EF1α*) to 29.55 (*SOD*) for the different tissues, from 12.39 (*RPL7A*) to 26.42 (*SOD*) under 20E treatments, and from 17.25 (*AK*) to 30.97 (*RPL28*) under the insecticide treatments (Figure 1). The average Ct values ranged from 13.63 (*RPL7A*) to 28.64 (*RPL28*) under the four experimental conditions. *RPL7A*, *EF1α*, and *AK* were the most abundant reference genes, whereas *RPL28* and *SOD* were the least expressed genes (Figure 1).

### 3.3. Stability of the Reference Genes under Different Experimental Conditions

The mRNA levels of nine candidate reference genes were described using their mean Ct values. To evaluate the stability of reference genes among four experiments, we compared the four algorithms NormFinder, BestKeeper, geNorm, and ΔCt method. Simultaneously, the overall stability ranking was calculated by RefFinder.

#### 3.3.1. Biotic Factors

**Figure 2:**
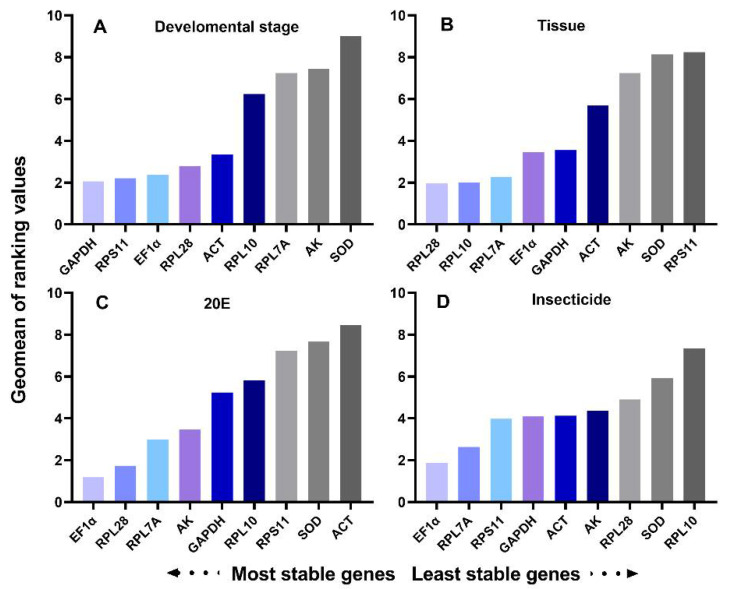
Expression stability of the candidate reference genes under four experimental conditions calculated by RefFinder: (**A**) developmental stages; (**B**) different tissues; (**C**) 20E treatment; (**D**) insecticide treatments. A lower Geomean order indicates more stable expression.

**Figure 3:**
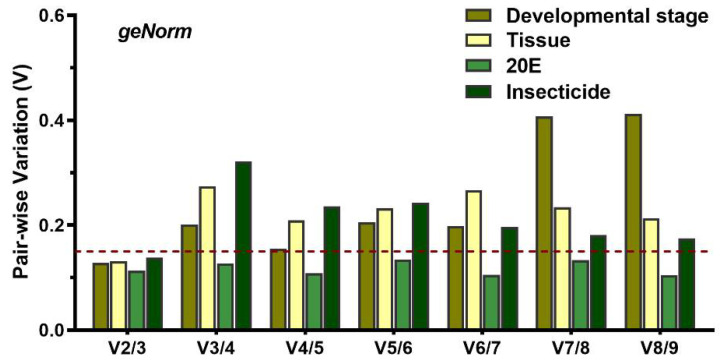
Determining the optimal number of reference genes for normalization by geNorm analysis. Average pair-wise variations (V_n_/V_n+1_) were calculated by geNorm between the normalization factors NF_n_ and NF_n+1_. A value above 0.15 indicates that an additional reference gene will significantly improve normalization.

Developmental stages: Expression analysis of each gene was performed on a complete sample set, including adults, pupae, and larval individuals at different developmental stages. All four programs identified that *SOD* as the least stable gene. *RPS11* was one of the most stable gene calculated by NormFinder and BestKeeper, while geNorm identified *GAPDH* as the most stable reference gene (Table 3). RefFinder ranked the genes from the most stable to the least stable as follows: *GAPDH* > *RPS11* > *EF1α* > *RPL28* > *ACT* > *RPL10* > *RPL7A* > *AK* > *SOD* (Figure 2). Pair-wise variation analysis for the six developmental stages indicated that the V_2/3_ values were less than 0.15 (Figure 3). The results suggested that *GAPDH* and *RPS11* were the best for normalization of developmental stage experiments.

Tissue: All three programs, except BestKeeper, showed that *RPL28* and *RPL10* were the most stable genes, and that *SOD* was the least stable gene. BestKeeper identified *RLP28* and *GAPDH* were the most stable gene. RefFinder showed a comprehensive ranking order from the most stable to the least stable gene: *RPL28* > *RPL10* > *RPL7A* > *EF1α* > *GAPDH* > *ACT* > *AK* > *SOD* > *RPS11* (Figure 2). GeNorm analysis suggested that the pair-wise variation value V_2/3_ was less than 0.15 (Figure 3). Thus, the two best reference genes (*RPL28* and *RPL10*) should be used to optimize gene expression studies of various tissues.

#### 3.3.2. Abiotic Stresses

20E treatment: After 20E induction in *T. absoluta* larva, geNorm and NormFinder identified *EF1α* and *RPL28* were the most stable reference genes. The ΔCt method and NormFinder showed that *RPL28* and *GAPDH* were the most stable genes, respectively. Using geNorm and NormFinder, *ACT* was the least stable gene for expression analysis in 20E induction, whereas the ΔCt method and BestKeeper ranked *SOD* and *RPL10* as least stable, respectively (Table 3). RefFinder showed the stability of nine reference genes decreased in the following order: *EF1α* > *RPL28* > *RPL7A* > *AK* > *GAPDH* > *RPL10* > *RPS11* > *SOD* > *ACT* (Figure 2). GeNorm analysis revealed that V_2/3_ was less than 0.15 (Figure 3). Thus, the two reference genes, *EF1α* and *RPL28*, were the best to normalize gene expression.

Insecticide treatments: Prior to the insecticide induction, we performed a bioassay to determine the LD_50_ dosage of four insecticides. The LD_50_ values for larva stressed by abamectin, spinosad, chlorantraniliprole, and indoxacarb were 23.838, 779.915, 0.259, and 336.704 mg/L, respectively (Table 4).

The mortality of the larvae in the control group, without insecticide stress, was less than 5%. The stability rankings produced by geNorm and NormFinder were similar, *EF1α* and *RPL7A* were the most stable genes, while *RPL10* was the least stable reference gene. BestKeeper identified *ACT* as the most stable reference gene. The ΔCt method showed that *RPL28* was the most stable reference gene (Table 3). According to the RefFinder results, the stability rankings were as follows: *EF1α* > *RPL7A* > *RPS11* > *GAPDH* > *ACT* > *AK* > *RPL28* > *SOD* > *RPL10* (Figure 2). GeNorm analysis showed that V_2/3_ was less than 0.15 (Figure 3). Thus, EF1α and RPL7A were the best reference genes for normalizing RT-qPCR data in the insecticide treatments.

### 3.4. Validation of Reference Genes with EcR

**Figure 4:**
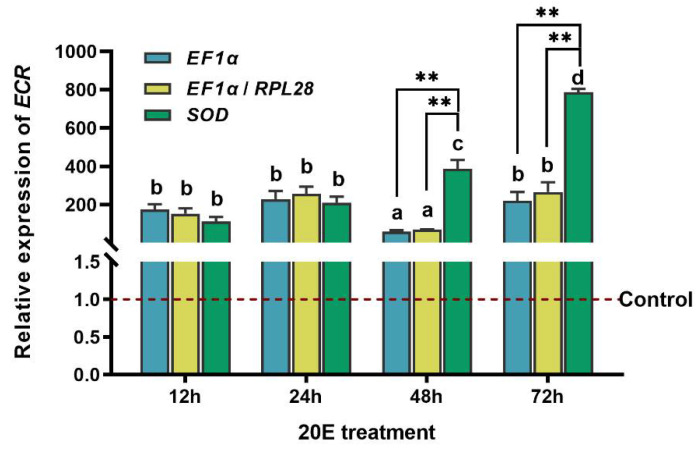
Validation of reference genes. Relative expression level of *EcR* was detected by RT-qPCR at 12, 24, 48, and 72 h after 20E treatment. Three reference genes combination (*EF1α*, *EF1α+RPL28*, *SOD*) were used for the normalization. The data are denoted as mean ± standard error (SE). Asterisk indicates that the expression of *EcR* normalized by each reference gene(s) was significantly different (*p* < 0.01, two-way ANOVA followed by Tukey’s multiple comparison test).

To assess the stability of selected reference genes, the expression level of *EcR* was analyzed after 20E treatment. The expression of *EcR* was compared using the following internal references, *EF1α*, *EF1α* and *RPL28* (most stable reference genes), and *SOD* (the least stable reference gene). The significant differences were evaluated by normalizing *EcR* expression in larvae stressed by 20E using the most stable reference genes and the least stable reference genes. Across 12, 24, 48, and 72 h after 20E treatments, *EcR* transcript levels increased significantly in 20E treatments compared with controls no matter whether it was normalized by the most stable reference gene (*EF1a*), the combination of the two most stable reference genes (*EF1a* and *RPL28*), or the least stable reference gene (*SOD*) (Figure 4). However, the expression level of *EcR* normalized by the most stable reference gene or the combination of the two best reference genes was significantly different from the expression level calculated using the least suitable reference gene among all treatments (*p* < 0.01). At 48 and 72 h after 20E treatment, normalization with the least stable reference gene *SOD* resulted in increased gene expression levels (Figure 4).

## 4. Discussion

RT-qPCR is the most extensively method for measurement of gene expression. However, its accuracy and reliability depend on stable reference genes for proper data normalization. To avoid data ambiguity, each candidate reference gene must be verified against certain experimental conditions. Research on reliable reference gene selection has been included in quantitative expression analysis of animals [25,26] and plants [27]. Our study is the first direct evaluation of the expression stability of candidate reference genes in *T. absoluta*. The main goal in this study was to identify stable reference genes among the candidate genes for use in accurate normalization of gene expression in *T. absoluta*. We used four different algorithms to assess their stabilities at different developmental stages, among tissues, and changes induced by 20E and insecticide treatments.

Two or more reference genes are often used for more accurate quantitative analysis. More than one reference gene was helpful to reduce the deviation of data normalization [11,12,13]. As there is no universal candidate reference gene that can be used for all conditions, the most suitable reference genes will vary with specific experiments [13,24]. Evaluation of primer efficiency must be conducted before gene expression analysis. In this study, all reference genes varied according to conditions, but *EF1α*, *RPL28*, and *GAPDH* seem to best fit the universality criteria of reference genes for *T. absoluta*. These genes may be the best first choices for standardization in gene expression analysis. Due to differences in program algorithms [8,28], we found that individual ranking of the tested reference genes varied when using different computational programs. 

In *Spodoptera exigua*, *β*-actin1 (*ACT1*), *β*-actin2 (*ACT2*), *SOD*, *EF1*, and *GAPDH* were stably expressed at different developmental stages [29]. In *Spodoptera litura*, *GAPDH* and *ubiquinol-cytochrome C reductase* (*UCCR*) were the best reference genes for developmental stages [30]. Among the different development stages, the optimal reference gene was *EF1* in *Plutella xylostella* [31]. Our research showed that *GAPDH* and *RPL28* were stably expressed at all developmental stages of *T. absoluta*. Several studies on reference genes have been conducted in tissues. *RPL10*, *elongation factor 2* (*EF2*), and *RPL17A* were highly ranked as reference genes in all tissues of *S. exigua* [29]. In *S. litura*, *RPL10*, *AK*, and *EF1* were the most appropriate reference genes for different tissues [30]. In *P. xylostella* tissues, the most suitable gene for internal control was *EF1* [31]. In the present study, we showed that *RPL28*, *RPL10,* and *GAPDH* comprised the best set of reference genes for *T. absoluta*. *GAPDH* plays an important role in energy metabolism and encodes an enzyme in the glycolytic pathway [29]. Stable expression of this gene appears common in lepidopteran insects such as *Helicoverpa armigera* [32] and *T. armoricanus* [12]. In the following gene expression analysis, the best combination was *GAPDH* and *RPL28*. Ribosomal protein (RP) is the main component of ribosomes and mainly function in the intracellular protein biosynthesis in cells. In this study, the expression stabilities of *RPL7A*, *RPL10*, *RPL28*, and *RPS11* in *T. absoluta* were evaluated under four different experimental conditions. The assessment of RP in insects suggests that RP is stable gene and can be used in gene expression studies. *RPL9* and *RPL10* were stable in different developmental stages, tissues, and temperature stresses of *Sogatella furcifera* [33].

Under different abiotic factors and experimental conditions, the best combinations of reference genes were as follows: *EF1α* and *RPL28* for 20E induction; *EF1α* and *RPL7A* for insecticide treatments. *EF1α* is vital for the translation of genes to proteins by catalyzing GTP-dependent binding of aminoacyl-tRNA to the receptor site of the ribosome [34,35]. This gene exhibited the most stable expression among various biotic and abiotic factors in insects. For example, *EF1α* was an optimal reference gene for normalization in *Agrilus planipennis* [36], *S. litura* [30], and *Rhodnius prolixus* [27], which also ranked *EF1α* as the most stable reference gene. However, *EF1α* was not an acceptable gene in *Bemisia tabaci* [37]. In addition, the gene that codes for ribosomal protein *RPL28* and *RPL7A* were the most suitable genes under certain conditions, such as insecticide treatments.

To validate our findings, we analyzed the expression of *EcR* in response to 20E stress. *EcR* is a direct response gene, and 20E treatment enhances the production of *EcR* transcripts. A heterodimer formed by the insect ecdysone receptor and ultraspiracle mediates the ecdysone signal [38]. It initiates the ecdysis cascade, including the expression of transcription factors so that the insect can successfully complete ecdysis and metamorphosis and acquire innate immunity [39]. At the start of the fifth-instar larvae of *Bombyx mori*, the rising steroid titers favor *EcR* expression [40]. In *S. exigua*, the relative mRNA expression of *SeEcR* after 20E injection was assayed using RT-qPCR. There was a burst in *SeEcR* transcription after 20E injection, which increased compared to the mRNA level in control insects [40]. In addition, results on ultraspiracle (*USP*), which is also the molecular target of ecdysone, showed that in the presence of 20E, the level of *PIUSP-2* expression peaked at 18 h [41]. Our results demonstrated that *EcR* was consistently highly expressed at 12, 24, 48, and 72 h after 20E stress in *T. absoluta* when the most stable genes were used for normalizing expression. However, the expression profile of *EcR* was significantly altered when an unstable reference gene was used for the normalization. Therefore, the selection of appropriate reference genes for normalization is important for accurate estimation of target gene expression. For the first time, this study laid a methodological foundation for the development of *T. absoluta* functional gene expression analysis.

## 5. Conclusions

In conclusion, nine candidate reference genes were studied using four dedicated algorithms, and *EF1α* and *RPL28* were found to be the most stable reference genes for *T. absoluta* at different stages, in various tissues, under 20E and insecticide treatments. These results provide reference gene standardization for RT-qPCR technology. Several potential references were identified to accurately assess the target gene expression profile in *T. absoluta*. The data presented here offers insight into expression profiling in gene functional studies in *T. absoluta*. This study documents the stability of a reference genes in *T. absoluta*. The information will help future studies that seek to understand how *T. absoluta* adapts to adverse environmental conditions.

## Figures and Tables

**Table 1:** GenBank accession numbers and functions of candidate reference genes.

Gene Symbol	Gene Name	Accession Number	(Putative) Function
*EF1α*	Elongation factor 1 α	MZ054826	Mediates recruitment of aminoacyl-transfer RNA to ribosome
*AK*	Arginine kinase	MZ054821	Key enzyme for cellular energy metabolism
*SOD*	Superoxide dismutase	MZ054825	Highly specific superoxide dismutase activity
*ACT*	*β*-actin	MZ054824	Cell motility, structure and integrity
*GAPDH*	Glyceraldehyde-3- Phosphate dehydrogenase	MZ054823	Glycolytic enzyme
*RPL7A*	Ribosomal protein L7A	MZ054828	Structural constituent of ribosome
*RPL10*	Ribosomal protein L10	MZ054827	Structural constituent of ribosome
*RPL28*	Ribosomal protein L28	MZ054829	Structural constituent of ribosome
*RPS11*	Ribosomal protein S11	MZ054830	Structural constituent of ribosome

**Table 2:** Primers of reference genes in *T. absoluta*.

Gene	Primer Sequence (5′–3′)	Product Size (bp)	E (%)	R^2^
*EF1α*	F: CCTGGGCACAGAGATTTCAT R: GATCAGCTGCTTGACACCAA	171	98.3	0.997
*AK*	F: GCCCAGTACAAGGAGATGGA R: ACCACACGAGGAAGGTCTTG	238	99.3	0.998
*SOD*	F: GGGCCTCATTTCATTGCTTA R: CTTCGCCACTGCTTATAGCC	190	109.1	0.996
*ACT*	F: GCGACATCAAGGAGAAGCTC R: CAAGCTTCCATACCCAGGAA	187	97.2	0.991
*GAPDH*	F: GCGTCAACCTTGAAGCCTAC R: TTACCAGAGGGACCGTCAAC	181	102	0.993
*RPL7A*	F: TCAACCAGTTCACCCAGACA R: CACGAGCTGAGCCTTCTTCT	227	100.8	0.995
*RPL10*	F: CTTCATCCCTTCCACGTCAT R: TGAAACCCCACTTCTTGGAC	250	95.6	0.998
*RPL28*	F: TCAGACGTGCTGAACACACA R: GCCAGTCTTGGACAACCATT	185	92.9	0.995
*RPS11*	F: AAGACCTGCCGATATGCAAC R: TAGCCGTAGTCTGAGCAGCA	156	98.4	0.994

E, RT-qPCR efficiency; R^2^, regression coefficient; F, forward primer; R, reverse primer.

**Table 3:** Ranking of the *T. absoluta* reference genes under four experimental conditions.

Biotic Conditions	Rank	geNorm		Normfinder		BestKeeper		ΔCt	
		Gene	Stability	Gene	Stability	Gene	Stability	Gene	Stability
Developmental stage	1	*GAPDH*	1.654	*RPS11*	0.110	*RPL28*	0.785	*RPL28*	0.889
	2	*EF1* *α*	1.669	*GAPDH*	0.189	*RPS11*	1.001	*RPL10*	0.916
	3	*RPS11*	1.776	*RPL28*	0.240	*GAPDH*	1.129	*ACT*	0.944
	4	*RPL28*	1.781	*EF1* *α*	0.576	*EF1α*	1.422	*EF1* *α*	0.976
	5	*ACT*	1.875	*ACT*	0.901	*ACT*	1.526	*RPS11*	0.979
	6	*RPL10*	2.078	*RPL10*	1.131	*AK*	1.643	*AK*	0.987
	7	*RPL7A*	2.354	*RPL7A*	1.424	*RPL10*	2.016	*RPL7A*	1.025
	8	*AK*	3.187	*AK*	1.845	*RPL7A*	2.386	*GAPDH*	1.031
	9	*SOD*	4.567	*SOD*	2.110	*SOD*	2.797	*SOD*	1.359
Tissues	1	*RPL28*	1.343	*RPL28*	0.235	*RPL28*	0.779	*RPL28*	0.825
	2	*RPL10*	1.348	*RPL10*	0.385	*GAPDH*	1.023	*RPL10*	0.912
	3	*RPL7A*	1.364	*RPL7A*	0.435	*RPL7A*	1.033	*ACT*	0.924
	4	*GAPDH*	1.668	*GAPDH*	0.772	*RPL10*	1.121	*RPS11*	0.955
	5	*ACT*	1.697	*ACT*	0.829	*EF1α*	1.206	*EF1α*	0.961
	6	*EF1* *α*	1.823	*EF1* *α*	1.013	*SOD*	1.250	*AK*	0.982
	7	*AK*	1.990	*AK*	1.106	*Actin*	1.503	*RPL7A*	1.013
	8	*RPS11*	2.184	*RPS11*	1.287	*AK*	1.656	*GAPDH*	1.032
	9	*SOD*	2.205	*SOD*	1.297	*RPS11*	1.909	*SOD*	1.264
20E	1	*EF1* *α*	0.651	*EF1* *α*	0.161	*GAPDH*	0.440	*RPL28*	0.946
	2	*RPL28*	0.710	*RPL28*	0.235	*RPL28*	0.496	*RPL10*	0.969
	3	*RPL7A*	0.741	*AK*	0.280	*AK*	0.510	*ACT*	0.973
	4	*AK*	0.753	*RPL7A*	0.342	*EF1α*	0.628	*RPS11*	0.991
	5	*RPS11*	0.852	*SOD*	0.473	*RPS11*	0.923	*EF1α*	0.995
	6	*SOD*	0.900	*RPS11*	0.493	*SOD*	1.234	*AK*	0.995
	7	*RPL10*	0.906	*RPL10*	0.502	*RPL7A*	1.316	*RPL7A*	1.003
	8	*GAPDH*	0.989	*GAPDH*	0.630	*ACT*	1.378	*GAPDH*	1.020
	9	*ACT*	0.989	*ACT*	0.630	*RPL10*	1.419	*SOD*	1.097
Insecticide	1	*EF1* *α*	1.303	*EF1* *α*	0.391	*ACT*	0.440	*RPL28*	0.819
	2	*RPL7A*	1.350	*RPL7A*	0.559	*RPL7A*	0.496	*RPS11*	0.959
	3	*RPS11*	1.474	*AK*	0.675	*EF1α*	0.510	*RPL10*	0.968
	4	*AK*	1.490	*RPS11*	0.734	*RPL10*	0.628	*ACT*	0.987
	5	*GAPDH*	1.545	*SOD*	0.795	*SOD*	0.923	*EF1α*	0.988
	6	*ACT*	1.558	*ACT*	0.844	*AK*	1.234	*AK*	0.990
	7	*SOD*	1.569	*GAPDH*	0.877	*RPS11*	1.316	*RPL7A*	1.005
	8	*RPL28*	1.728	*RPL28*	1.054	*GAPDH*	1.378	*GAPDH*	1.057
	9	*RPL10*	1.755	*RPL10*	1.057	*RPL28*	1.419	*SOD*	1.106

**Table 4:** Toxicity of four insecticides to third-instar larvae of *T. absoluta*.

Insecticides	N ^a^	Slope ± SE ^b^	LD_50_ (95% CL) ^c^ mg/L	χ^2 d^
Abamectin	300	2.167 ± 0.242	23.838 (20.021–28.819)	0.981
Spinosad	300	0.880 ± 0.089	779.915 (504.427–1227.212)	0.348
Chlorantraniliprole	300	1.824 ± 0.211	0.259 (0.187–0.366)	1.990
Indoxacarb	300	0.976 ± 0.131	336.704 (188.020–642.933)	1.739

^a^ The total number of tested individuals. ^b^ SE = standard error. ^c^ 95% CL = 95% confidence limit. ^d^ Chi-square testing linearity of dose-mortality responses.

## Data Availability

The data presented in this study are available in article.

## Supplementary Materials

The following are available online at https://www.mdpi.com/article/10.3390/insects12070589/s1, Figure S1: Melting curves of nine candidate reference genes show single peaks.
