# Supplementary figures and images for: Selection and Validation of Reference Genes for Gene Expression Analysis in Tuta absoluta Meyrick (Lepidoptera: Gelechiidae)
Source: Insects. 2021 Jun 29;12(7):589. doi: 10.3390/insects12070589 (PMC8305163; doi:10.3390/insects12070589)

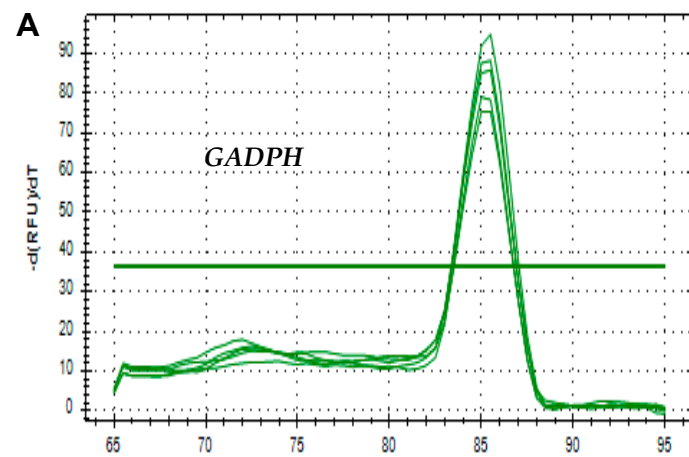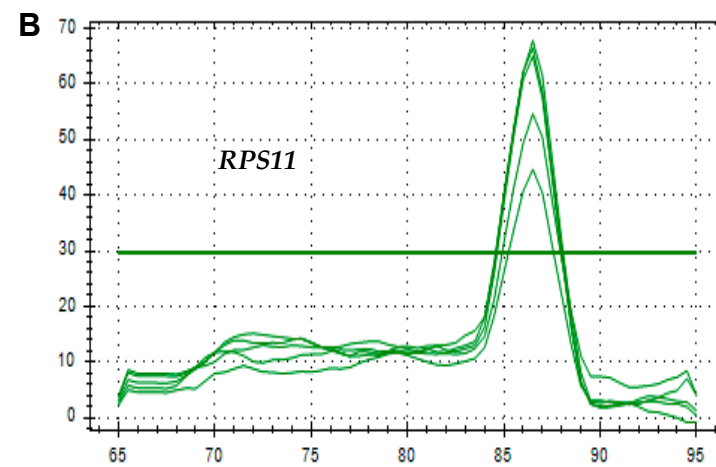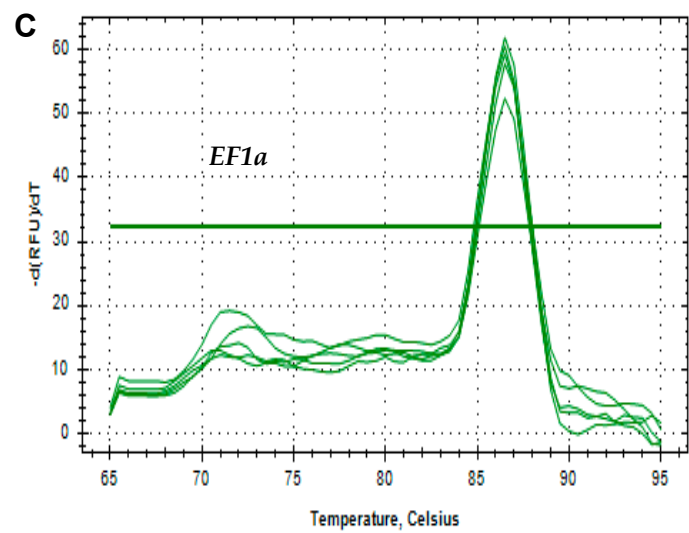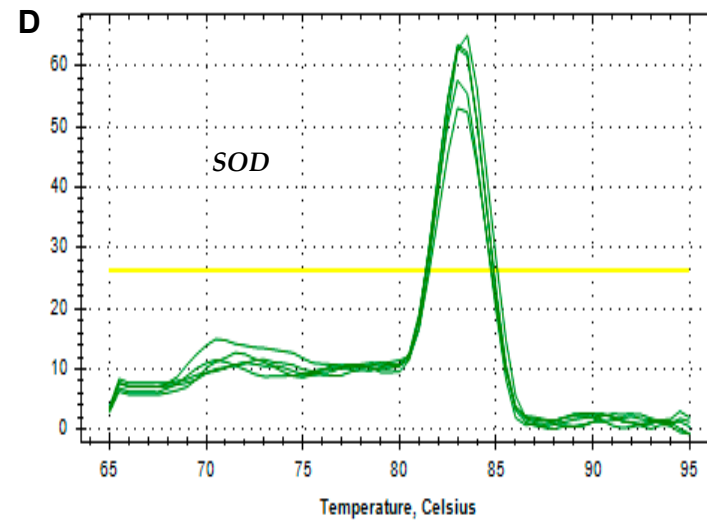

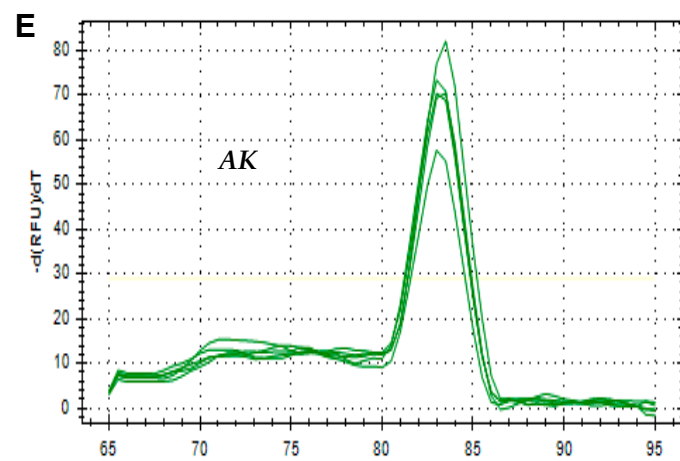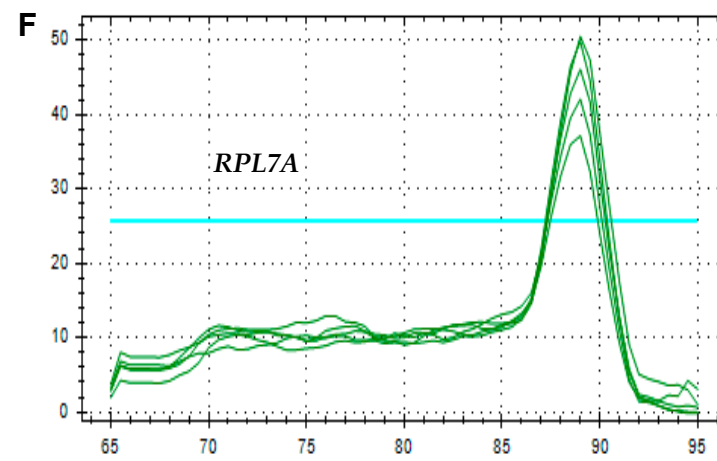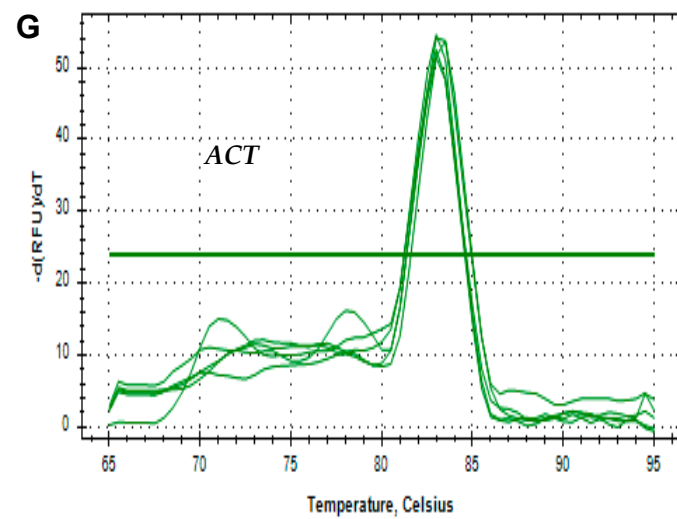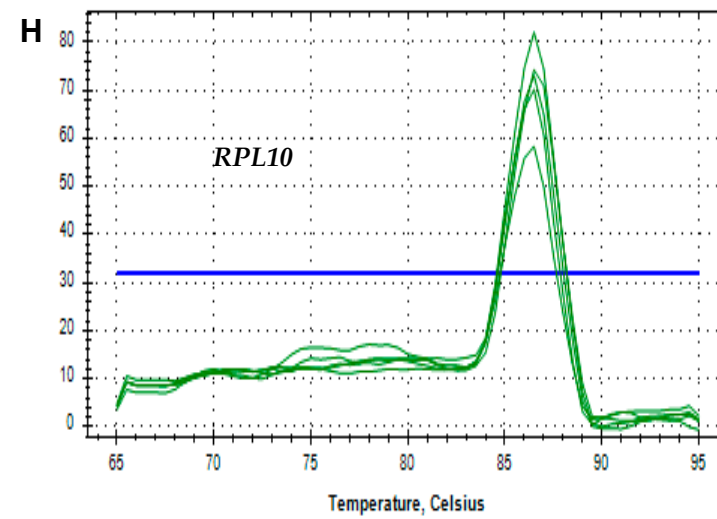

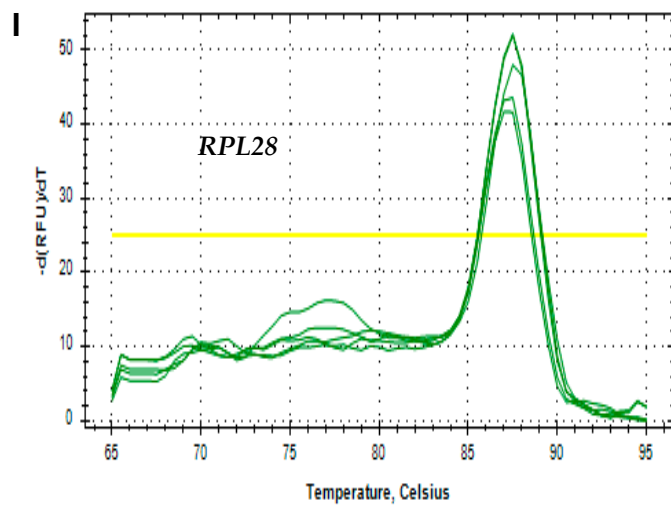

Figure S1. Melting curves of nine candidate reference genes show single peaks.

Supplement: Supplementary file 1 [file insects-12-00589-s001.zip › insects-1250481-SI.pdf]
